# Guidewire exchange vs new-site placement for temporary dialysis catheters insertion in ICU patients: is there a greater risk of colonization or dysfunction?

**DOI:** 10.1186/2197-425X-3-S1-A463

**Published:** 2015-10-01

**Authors:** E Coupez, J-F Timsit, A Boyer, L Bouadma, E Canet, K Klouche, L Argaud, J Bohé, M Garrouste-Orgeas, C Mariat, F Vincent, S Cayot, O Cointault, A Lepape, M Darmon, S Ruckly, C Schwebel, A Lautrette, B Souweine

**Affiliations:** Medical Intensive Care Unit, University Hospital of Clermont-Ferrand, Clermont-Ferrand, France; Medical Intensive Care Unit, Albert Michallon Teaching Hospital, University Hospital of Grenoble, Grenoble, France; U 823, Outcome of Cancers and Critical Illness', Albert Bonniot Institute, La Tronche, France; Medical Intensive Care Unit, Pellegrin Teaching Hospital, University Hospital of Bordeaux, Bordeaux, France; Medical Intensive Care Unit, Bichat-Claude Bernard Teaching Hospital, Paris, France; Medical Intensive Care Unit, Saint Louis Teaching Hospital, Paris, France; Medical Intensive Care Unit, Lapeyronie Teaching Hospital, University Hospital of Montpellier, Montpellier, France; Medical Intensive Care Unit, Edouard Herriot Teaching Hospital, University of Lyon, Lyon, France; Medical Intensive Care Unit, Pierre Benite Teaching Hospital, University Hospital of Lyon, Lyon, France; Critical Care Medicine Unit, Saint-Joseph Hospital, Paris, France; Nephrology and Critical Care Unit, Nord Teaching Hospital, University of Saint-Etienne, Saint-Etienne, France; Medical Intensive Care Unit, Avicenne Teaching Hospital, Paris, France; Department of Anaesthesiology and Critical Care Medicine, University Hospital of Clermont-Ferrand, Clermont-Ferrand, France; Nephrology and Critical Care Medicine, Rangueil Teaching Hospital, University of Toulouse, Toulouse, France; Department of Anaesthesiology and Critical Care Medicine, Pierre Benite Teaching Hospital, University Hospital of Lyon, Lyon, France; Medical Intensive Care Unit, Nord Teaching Hospital, University of Saint-Etienne, Saint-Etienne, France; LMGE, Laboratoire Micro-Organismes, Génôme et Environnement, UMR CNRS 6023, Clermont-Ferrand University, Clermont-Ferrand, France

## Introduction

Critically ill patients routinely require temporary dialysis catheters (DCs) for renal replacement therapy (RRT). They carry a high risk for developing end-stage renal disease. Though, their vascular accesses must be preserved. Guidewire exchange (GWE) is often use to avoid venipuncture at new site. However, the impact of GWE on infection and dysfunction of DC in ICU has never been studied.

## Objectives

The aim of this study was to compare the effect GWE and new-site placement (NSP) strategies on DC colonization and dysfunction in patients requiring DC placement.

## Methods

Using data from the ELVIS RCT (1496 critically ill adults requiring DC for RRT or plasma exchange) we performed a matched- cohort analysis. Cases were DCs inserted by GWE (N = 178) (first DC inserted by GWE in patients with multiple DC inserted by GWE), controls were DCs inserted by NSP (N = 178). We matched each case with a control based on the following criteria: participating center, SAPS II +/-10, insertion site (jugular or femoral), side for jugular site, and duration between ICU admission and DC placement. DC colonization was defined by a quantitative DC-tip culture yielding ≥1000 CFU/mL with vortexing and ≥100 CFU/mL with sonication, and when DC was left in place at ICU discharge, by a positive blood culture drawn from the DC hub. DC dysfunction was defined as DC removal as a result of inadequate catheter flow despite attempts to restore DC patency.

A sensitivity analysis was performed on the specific subgroup of patients with a DC changed because of dysfunction of the previous one. (138 GWE-DCs and 160 NSP-DCs).

The effect of the strategy of catheter insertion (GWE vs NSP) on DC colonization and dysfunction was estimated using a marginal Cox model.

## Results

In the matched-cohort analysis, GWE-DC and NSP-DC patients were not different for gender (118 (66.3%) vs. 109 (61.2%) male) and illness severity on admission (mean SAPS II: 66 vs. 66). However, GWE patients were younger (63 vs 67 years; P = 0.05) had longer median ICU length of stay (19 vs. 17 days; P = 0.004) and median hospital length of stay (37.5 vs. 32.5 days; P = 0.02). Between GWE-DCs and NSP-DCs, there was no difference in DC colonization (10 (5.6%) vs 10 (5.6%); hazard ratio (HR), 1.68 (0.40-6.98); P = 0.48) but DC dysfunction was more frequent (67 (37.6%) vs 28 (15.7%); HR, 3.68 (2.07-6.49); P < 0.0001), respectively. Major DC infection was observed in 2 GWE-DC and in 1 NSP-DC patients. In the sensitive analysis after adjustment on insertion site and side placement, GWE-DC and left side placement were independently associated with dysfunction; HR, 2.48 (1.67-3.68); P < 0.0001; HR, 2.06 (1.42-2.99); P = 0.0001, respectively.

## Conclusions

In ICU patients, as compared to NSP, GWE of DCs did not contribute to DC colonization infection but is associated with a more than 2 fold increase of DC dysfunction.

